# TRAF6 Promotes Gastric Cancer Cell Self-Renewal, Proliferation, and Migration

**DOI:** 10.1155/2020/3296192

**Published:** 2020-07-17

**Authors:** Mengting Yang, Meng Jin, Kailong Li, Haifeng Liu, Xiaxia Yang, Xiaobei Zhang, Bin Zhang, Aihua Gong, Qingli Bie

**Affiliations:** ^1^Department of Laboratory Medicine, Affiliated Hospital of Jining Medical University, Jining Medical University, Jining, Shandong, China; ^2^Key Laboratory of Laboratory Medicine of Jiangsu Province, School of Medicine, Jiangsu University, Zhenjiang, Jiangsu, China; ^3^Department of General Surgery, Affiliated Hospital of Jining Medical University, Jining, Shandong, China; ^4^Department of Ultrasound, Affiliated Hospital of Jining Medical University, Jining, Shandong, China; ^5^Department of Central Laboratory, Affiliated Hospital of Jining Medical University, Jining, Shandong, China

## Abstract

Gastric cancer is the third most common type of tumor associated with death. TRAF6 belongs to the tumor necrosis factor receptor-associated factor family and has been demonstrated to be involved in tumor progression in various cancers. However, the exact effect of TRAF6 on gastric cancer stem cells has not been extensively studied. In this study, abnormal expression of TRAF6 was found in gastric cancer tissues. Overexpression of TRAF6 enhanced proliferation and migration, and TRAF6 knockdown reversed this phenomenon in gastric cancer cells. Moreover, TRAF6 may inhibit differentiation and promote stemness and epithelial-mesenchymal transition (EMT). Transcriptome profiles revealed 701 differentially expressed genes in the wild-type group and the TRAF6 knockout group. Potential molecules associated with cell proliferation and migration were identified, including MAPK, FOXO, and IL-17. In conclusion, TRAF6 is a significant factor promoting proliferation and migration in gastric cancer cells and may provide a new target for the accurate treatment of gastric cancer.

## 1. Introduction

Although the prevalence of gastric cancer has declined worldwide since the middle of the last century, it remains the fifth most common malignant tumor and the third most common cause of death among tumor types [1]. The main therapy for patients with gastric cancer is surgical resection and adequate lymphadenectomy, which may cause patient suffering [2]. Therefore, the identification and exploration of novel targets involved in gastric progression are urgently required.

Studies have revealed that tumor necrosis factor receptor-associated factor (TRAF) proteins inhibit TRAF signaling by blocking the interaction between TRAF receptors and short peptides or small molecules [3]. The TRAF family has many members, including TRAF6, which has been regarded as a key factor in innate immune response. As an E3 ubiquitin ligase, TRAF6 may rely on ubiquitin to regulate tumorigenesis [4]. TRAF6 is a significant oncogene in pancreatic cancer [5], prostate cancer [6], and nasopharyngeal carcinoma [7]. In addition, TRAF6 activates NF-*κ*B signaling in RAS-driven lung cancers [8]. Furthermore, it promotes tumor development by promoting AKT ubiquitination and phosphorylation in oral and breast cancers [9].

TRAF6 overexpression and knockdown experiments in the present study revealed that TRAF6 is highly expressed in gastric cancer tissues and promotes proliferation, migration, and stemness. Moreover, we demonstrated that FOXO, MAPK, and IL-17 signaling pathways are involved in the regulation of proliferation, migration, and stemness due to TRAF6 in gastric cancer cells.

## 2. Materials and Methods

### 2.1. Cell Culture and Reagents

The gastric cancer cell lines MGC-803 and HGC-27 were obtained from the American Type Culture Collection (ATCC). MGC-803 and HGC-27 cells were cultured in high-glucose Dulbecco's modified Eagle's medium (DMEM; Gibco) with 10% fetal bovine serum (FBS, Gibco) in standard conditions (37°C and 5% CO_2_ in a humidified incubator).

### 2.2. Ethics Statement

The study conformed to principles outlined in the Declaration of Helsinki and was conducted in accordance with approved guidelines. Written informed consent was obtained from each participant prior to the collection of tissue samples. All samples were acquired in accordance with the regulations and approval of the Medical Ethics Committee of Jining Medical University.

### 2.3. siRNA Transfection

TRAF6 siRNA samples and controls were provided by GenePharma (Shanghai, China). The sequences of TRAF6 siRNA are listed in Table 1. Briefly, 1 × 10^5^ cells were cultured in six-well plates, and Lipofectamine 2000 transfection reagent was used to perform transfections during the following days. After incubation for another 48 h, transfected cells were collected for further experiments.

### 2.4. Real-Time Quantitative Polymerase Chain Reaction Analysis

RNAiso Plus (Takara, Beijing, China) was used to isolate total RNA from tumor cells. According to the manufacturer's instructions for reverse transcription, cDNA was synthetized and used as a template with *β*-actin as the endogenous control for real-time polymerase chain reaction (RT-PCR). RT-PCR primer sequences are listed in Table 2.

### 2.5. Transwell Migration Assay

Tumor cell migration was assessed using 8 mm aperture chamber inserts (BD Biosciences, Franklin Lakes, NJ, USA). A total of 1 × 10^5^ cells were cultured in the upper chamber in a 24-well plate and were able to migrate toward the medium containing 10% FBS. After 18 h of culture, cells were fixed with methanol, washed with PBS, and then stained with crystal violet. The number of violet cells was counted using a microscope (Nikon, Japan).

### 2.6. Western Blot Assay

Proteins from tissues or cells were lysed using the radioimmunoprecipitation assay buffer including a protease inhibitor and then centrifuged at 4°C at 12000 g/min for 10 min and heated at 100°C for 10 min. After adding 10% SDS-PAGE, the protein was separated, and it was transferred to a polyvinylidene fluoride membrane, blocked using blocking solution for 2 h, and incubated with antibodies overnight at 4°C. The antibodies were GAPDH (CWBIO, China), TRAF6 (CWBIO, China), PCNA (Cell Signaling Technology, USA), LC3 (Cell Signaling Technology, USA), Sox2 (Cell Signaling Technology, USA), E-cadherin (Cell Signaling Technology, USA), and N-cadherin (Cell Signaling Technology, USA). Appropriate secondary antibodies were added, and the membrane was washed using tris-buffered saline and Tween. Finally, proteins were observed using a Tanon-5800 system.

### 2.7. Cell Counting Kit-8 Assay

Cells were seeded in 96-well plates at a density of 2000 cells per well and incubated at 37°C for 72 h. Each well was supplemented with 10 *μ*L of Cell Counting Kit-8 (CCK8) solution, followed by incubation for 2 h. We measured the OD value at 450 nm to assess cell proliferation.

### 2.8. Tumorsphere-Forming Assay

Transfected cells were cultured in a six-well plate with 5,000 or 10,000 cells per well in 2 mL of DMEM/F-12 medium, including 20 ng/mL EGF, 10 ng/mL bFGF, and 2% B27 (Sigma and Gibco). After 1 week, the number of spheroids was counted through light microscopy.

### 2.9. Colony-Formation Assay

Transfected cells were cultured in six-well plates with 2000 or 4000 cells per well and incubated at 37°C for 1 week until large cell clumps were observed. The medium was changed every 3 days. Colonies were fixed using 4% paraformaldehyde at 4°C for 30 min, washed using PBS, stained with 0.1% crystal violet for 20 min, and then observed using a microscope to count the numbers of colonies.

### 2.10. Transcriptome Sequencing

The RNA concentration and quality of HGC-27 cells in the control group and TRAF6 siRNA-2 group were assessed using a Nanodrop 2000 (Thermo Fisher Scientific, USA) and an Agilent 2100 Bioanalyzer (Agilent). Total RNA was sequenced using a HiSeqTM 2500 (Illumina) by Oebiotec (Shanghai, China).

### 2.11. Statistical Analysis

Experimental data are expressed as the mean ± standard deviation (SD). Data analysis was performed using Prism 8.0 software (GraphPad, San Diego, USA). The significance of group differences was tested using one-way analysis of variance and *t*-test, with *p* < 0.05 considered significant.

## 3. Results

### 3.1. TRAF6 Expression Was Upregulated in Gastric Tumor Tissues

**Figure 1:**
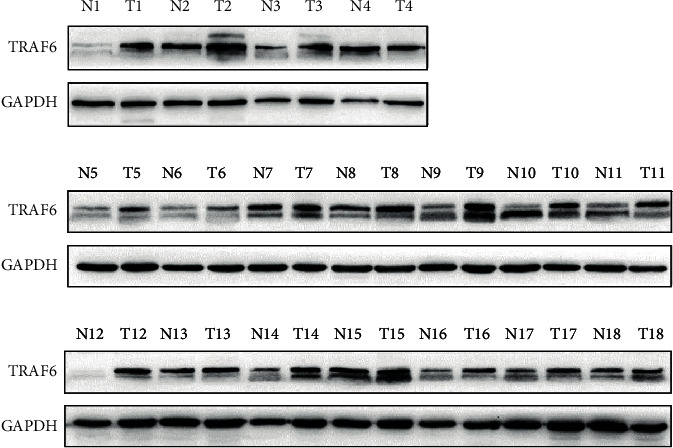
Western blot assay of TRAF6 protein levels in gastric cancer (T) and paired adjacent (N) tissues.

A total of 18 clinical gastric tumor tissue samples and paired adjacent tissues were obtained to test the expression of TRAF6. Results showed that TRAF6 presented two bands and was expressed significantly higher in gastric tumor tissues than in normal tissues (Figure 1). Pham et al. found that TRAF6 can be posttranscriptionally modified by SUMO-1 at lysines 124, 142, and 453 [10]. So we suspected that TRAF6 can be posttranscriptionally modified and molecular weight of TRAF6 may change in complicated cancer tissues. Thus, TRAF6 might play a significant role in the cell cycle and be associated with gastric cancer genesis and development.

### 3.2. Overexpression of TRAF6 Promoted Proliferation and Migration of Gastric Cancer Cells

**Figure 2:**
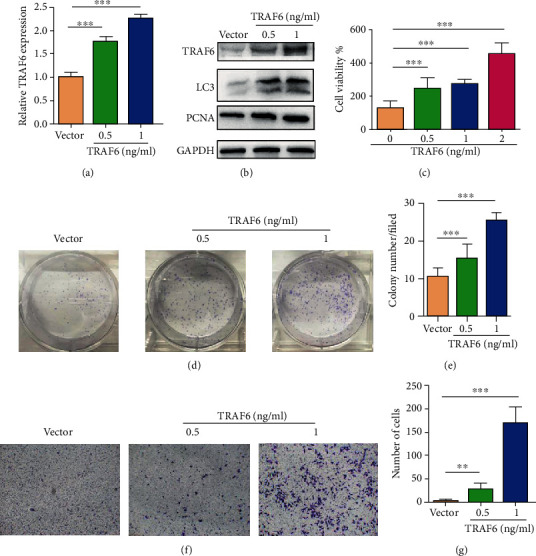
Overexpression of TRAF6 promoted cell proliferation and migration in HGC-27 gastric cancer cells. (a) Real-time PCR revealed TRAF6 expression in transfected vector and TRAF6 plasmid HGC-27 cells (*n* = 3, ^∗∗∗^*p* < .0001). (b) Western blot was used to confirm the expressions of TRAF6, PCNA, and LC3 in transfected vector and TRAF6 plasmid HGC-27 cells. (c) CCK8 assay for transfected vector and TRAF6 plasmid HGC-27 cells; cells were incubated at 37°C for 72 h. (d) Representative images of colony formation for transfected vector and TRAF6 plasmid HGC-27 cells and cells were cultured for 1 week. (e) Number of clones in (d) (*n* = 3, ^∗∗∗^*p* < .0001). (f) Transwell migration assay for transfected vector and TRAF6 plasmid HGC-27 cells. (g) Number of migrating cells in (f) (*n* = 3, ^∗∗∗^*p* < .0001).

To determine the role of TRAF6 in gastric cancer cells, HGC-27 cells were transfected with a vector or TRAF6 plasmids. Results suggested that TRAF6 expression was higher in the TRAF6 group than in the vector group (Figures 2(a) and 2(b)). Moreover, we analyzed the expression of PCNA and LC3 proteins and found that transfected TRAF6 promoted the expressions of these proteins (Figure 2(b)), which indicated that TRAF6 may promote the growth of HGC-27 cells. Then, colony formation and CCK8 assays were used to determine growth ability, with results indicating that TRAF6 promoted the proliferation of HGC-27 cells (Figures 2(c)–2(e)). To further investigate the effect of TRAF6 on cell migration, we performed the transwell chamber assay, which revealed that the number of passed cells in the TRAF6 group was significantly higher than that in the vector group (Figures 2(f) and 2(g)). In summary, overexpression of TRAF6 promoted the proliferation and migration of gastric cancer cells.

### 3.3. Suppression of TRAF6 Inhibited the Proliferation and Migration of Gastric Cancer Cells

**Figure 3:**
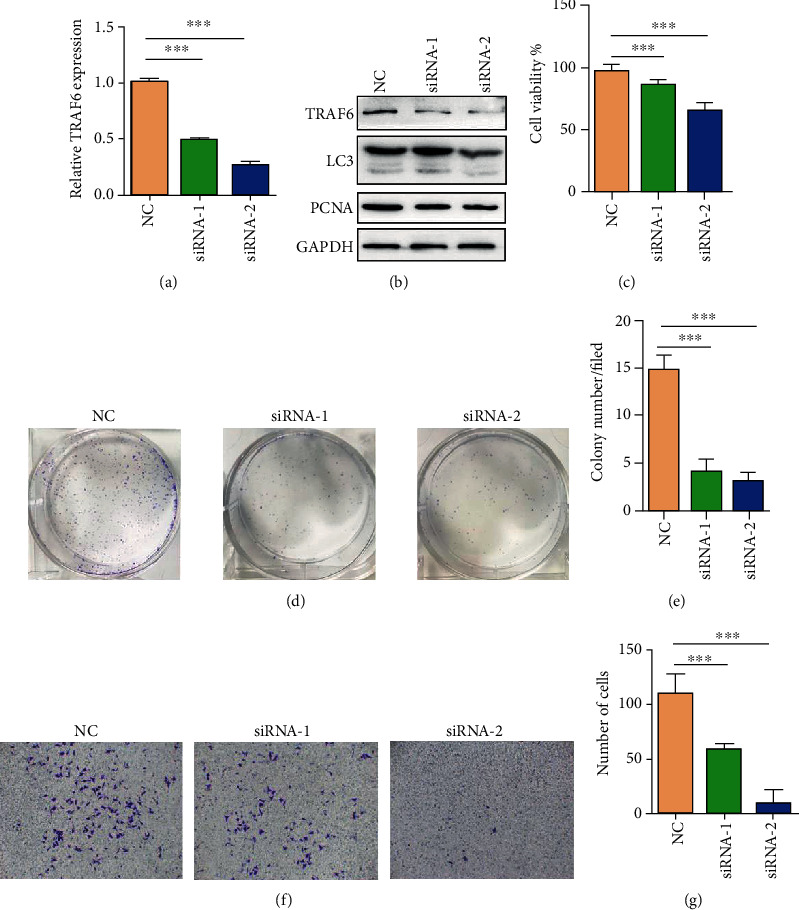
Knockdown of TRAF6 inhibited cell proliferation and migration in HGC-27 gastric cancer cells. (a) Real-time PCR revealed TRAF6 expression in transfected NC and TRAF6 knockdown HGC-27 cells (*n* = 3, ^∗∗∗^*p* < .0001). (b) Western blot was used to confirm the expressions of TRAF6, PCNA, and LC3 in transfected NC and TRAF6 siRNA-transfected HGC-27 cells. (c) CCK8 assay for transfected NC and TRAF6 siRNA-transfected HGC-27 cells; cells were incubated at 37°C for 72 h. (d) Representative images of colony formation for transfected NC and TRAF6 siRNA-transfected HGC-27 cells; cells were cultured for 1 week. (e) Number of clones in (d) (*n* = 3, ^∗∗∗^*p* < .0001). (f) Transwell migration assay for transfected NC and TRAF6 siRNA-transfected HGC-27 cells. (g) Number of migrating cells in (f) (*n* = 3, ^∗∗∗^*p* < .0001).

To further investigate the role of TRAF6 in gastric cancer, we knocked down the expression of TRAF6 by using siRNA. Protein and mRNA levels were considerably downregulated after TRAF6 knockdown in HGC-27 cells (Figures 3(a) and 3(b)). Moreover, compared with control cells, the expression of LC3 and PCNA decreased in TRAF6 knockdown in HGC-27 cells (Figure 3(b)). Colony formation and CCK8 assays were then used to investigate the role of TRAF6 in the proliferation of HGC-27 cells (Figures 3(c)–3(e)), and a transwell assay was used to explore their migration (Figures 3(f) and 3(g)). These results revealed that TRAF6 suppression markedly inhibited the cell proliferation and migration of gastric cancer cells. Overall, results shown in Figure 2 suggested that TRAF6 plays a key role in the proliferation and migration of gastric cancer cells.

### 3.4. TRAF6 Promoted Tumor Stemness and Epithelial Differentiation

**Figure 4:**
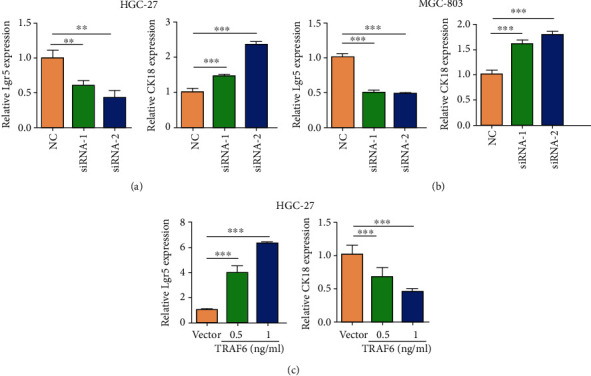
Effect of TRAF6 on differentiation and stemness markers in gastric cancer cells. (a) Real-time PCR analysis for the differentiated gastric epithelial marker CK18 and the cancer stem-like cell marker Lgr5 in NC and TRAF6 siRNA-transfected HGC-27 cells (*n* = 3, ^∗∗^*p* < .005; *n* = 3, ^∗∗∗^*p* < .00001). (b) Real-time PCR assay for the expression of CK18 and Lgr5 in NC and TRAF6 siRNA-transfected MGC-803 cells (*n* = 3, ^∗∗∗^*p* < .001; *n* = 3, ^∗∗∗^*p* < .001). (c) Real-time PCR assay for the expression of Lgr5 and CK18 in vector and TRAF6 plasmid-transfected HGC-27 cells (*n* = 3, ^∗∗∗^*p* < .00001; *n* = 3, ^∗∗∗^*p* < .00001).

**Figure 5:**
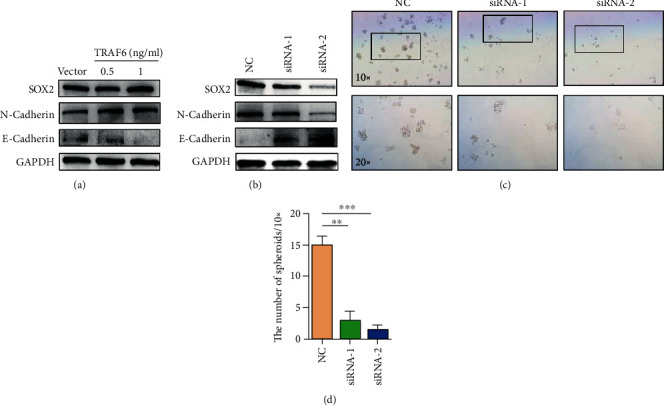
TRAF6 promoted stemness and inhibited differentiation in gastric cancer cells. (a) Western blot assay for the expressions of SOX2, N-cadherin, and E-cadherin in vector and TRAF6 plasmid-transfected HGC-27 cells. (b) Western blot assay for the expression of SOX2, N-cadherin, and E-cadherin in transfected NC and TRAF6 siRNA-transfected HGC-27 cells. (c) Mammospheres generated from single-cell cultures of NC and TRAF6 siRNA-transfected HGC-27 cells, imaged after 6 days of suspension culture (SF medium). (d) Number of mammospheres in (c) (mean ± SD numbers of spheres; *n* = 3, ^∗∗^*p* < .005).

Results confirmed that TRAF6 promoted the proliferation and migration of gastric cancer cells. The relationship of TRAF6 expression with differentiation and stemness properties was also demonstrated. The cancer stem-like cell marker Lgr5 and differentiation marker CK18 were detected using RT-PCR in HGC-27 and MGC-803 cells; TRAF6 knockdown resulted in the downregulation of Lgr5 and upregulation of CK18 (Figures 4(a) and 4(b)). By contrast, Lgr5 expression was significantly higher and CK18 was lower in TRAF6 plasmid-transfected HGC-27 cells than in the vector group (Figure 4(c)). As expected, the protein expressions of the stemness marker SOX2 and mesenchymal marker N-cadherin were upregulated, whereas the epithelial marker E-cadherin was downregulated in HGC-27 cells overexpressed with TRAF6 (Figure 5(a)). These results were reversed in the TRAF6 knockdown group (Figure 5(b)). Furthermore, suppression of TRAF6 inhibited the formation of spheroids in HGC-27 cells (Figures 5(c) and 5(d)). In conclusion, TRAF6 enhanced stemness but reduced differentiation; moreover, TRAF6 may promote EMT in gastric cancer cells.

### 3.5. Control and TRAF6 Knockdown Groups Exhibited Different Gene Expression Profiles in HGC-27 Cells

Control cells (NC) and HGC-27 cells transfected with siRNA-2 with high interference efficiency were analyzed using RNA-seq. The signature count was used to count the readings for each gene. After discarding reading segments for multiple genes and normalizing reading segments, 15261 genes were detected. When log_2_ fold change (FC) > 0.58 and *p* < 0.05, edgeR was used to identify differentially expressed genes (DEGs).

**Figure 6:**
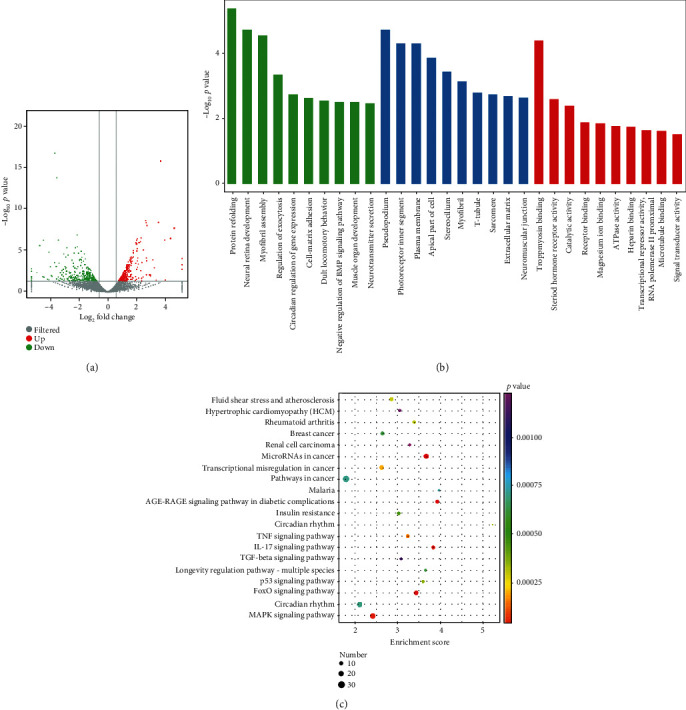
The GO enrichment and KEGG pathways analysis. (a) The *X*-axis represents log_2_ of the FC, and the *Y*-axis represents log_10_ of the *p* value. Genes with FC > 0.58 and *p* < 0.05 are represented by green and red dots. The red dots on the right indicate upregulated genes, and the green dots on the left indicate downregulated genes. Gray spots are filtered genes. (b) Ten key biological processes, molecular functions, and cellular components from GO enrichment analysis. The *Y*-axis represents negative log_10_ of the *p* value. (c) Ten key KEGG pathways. The *X*-axis represents enrichment score, and the *Y*-axis represents the pathway enrichment. Different colors represent different *p* values, and the size of the dot represents the number of genes.

Based on the edgeR standard, 701 DEGs were detected in NC and TRAF6 siRNA-2 groups, of which 373 (53.2%) were upregulated and 328 (46.8%) were downregulated. These two samples exhibited a dispersed distribution. The volcano map in Figure 6(a) shows the distribution of two different genomes.

### 3.6. Gene Ontology Enrichment Analysis and KEGG Enrichment Analysis

**Figure 7:**
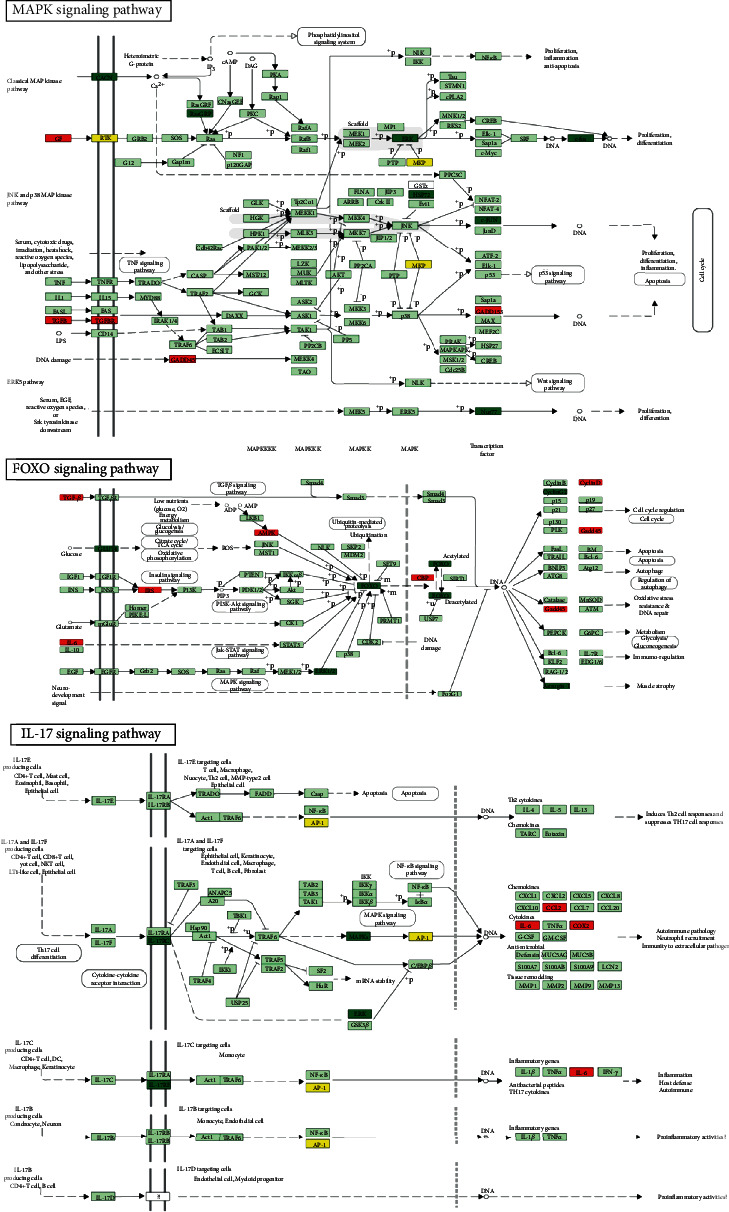
MAPK signaling, FOXO signaling, and IL-17 signaling associated with cell migration and proliferation. The red boxes represent upregulated genes, and the green boxes represent down-regulated genes.

Gene ontology (GO) enrichment analysis in the NC and TRAF6 siRNA-2 groups was performed to explore possible mechanisms related to migration, proliferation, and stemness in gastric cancer cells. Ten key biological processes, molecular functions, and cellular components were selected from GO enrichment (Figure 6(b)). KEGG analysis revealed important signaling pathways and biological functions, among which the MAPK and FOXO signaling pathways exhibited the most significant differences (Figure 6(c)). Based on KEGG analysis and experimental results, three signaling pathways associated with cell migration, proliferation, and stemness were identified. Genes related to proliferation and migration were almost all differentially involved in MAPK and FOXO signaling pathways. The CACN, ERK, and c-fos genes were downregulated in the conventional MAPK pathway. In the FOXO signaling pathway, FOXO and ERK1/2 genes were downregulated, whereas TGF-*β*, AMPK, and IRS genes were upregulated. In the IL-17 signaling pathway, IL-17RC, MAPK, and ERK genes were downregulated, whereas IL-6 and COX2 genes were upregulated (Figure 7).

## 4. Discussion

TRAF6 has been reported to be a significant oncogene in pancreatic cancer [5], prostate cancer [6], and nasopharyngeal carcinoma [7]. Moreover, TRAF6 is targeted in multiple immune functions through regulation of MAPK and NF-*κ*B activation [11–13]. In the present study, we revealed that TRAF6 plays a key role in gastric cancer. Crucially, overexpression of TRAF6 was confirmed to promote proliferation and migration, and TRAF6 knockdown reversed this phenomenon in gastric cancer cells. Furthermore, these results demonstrated that TRAF6 might be associated with the maintenance and generation of gastric cancer stem cells. We selected the control group and TRAF6 group with high interference efficiency to conduct RNA-seq, which measures transcription levels and their subtypes more comprehensively than do other methods [14]. Based on RNA-seq results, we suspected that MAPK, FOXO, and IL-17 signaling pathways can be associated with the promotion of growth and migration by TRAF6 in gastric cancer cells.

The MAPK pathway is believed to regulate the development and progression of cancer [15]. Moreover, MAPK signaling may be associated with proliferation, migration, and apoptosis in cancer cells [16–18]. ERK is one of the three major MAPK cascades, and ERK-mediated MAPK signaling activation has been reported in cervical cancer [19, 20]. The relationship between ERK activation and the progression of colorectal and breast cancers has also been studied [21–23]. In the present study, ERK was downregulated in the MAPK signaling pathway for the TRAF6 knockdown group. The cellular functions of FOXOs include cellular differentiation, apoptosis, and cell proliferation [24, 25]. Evidence has suggested that a disorder of the FOXO protein is crucial to the cell biology of cancer progression and tumorigenesis [26, 27]. FOXOs promote cell proliferation, survival, and invasion in breast and colon cancers [28–31]. TRAF6 depends on lysine 63 (K63) to activate ubiquitination [4], and FOXOs' promotion of cell proliferation is regulated by ubiquitin proteasome pathways [32]. TRAF6 was confirmed to regulate stromal cell proliferation through the Akt/mTOR signaling pathway in prostatic hyperplasia [33]. Furthermore, in oral and breast cancers, the underlying molecular mechanism of TRAF6 is the promotion of AKT ubiquitination and phosphorylation [9]. Micro-RNAs play a key role in the regulation of FOXOs in breast cancer [34], classical Hodgkin's lymphoma [35], endometrial cancer [36], osteosarcoma [37], prostate cancer [38], and lung cancer [39]. According to KEGG analysis, micro-RNAs in cancer were also highly correlated. TRAF6 may regulate proliferation and migration of gastric cancer cells through the FOXO signaling pathway. IL-17 also activates the MAPK pathway [40], but mechanisms that activate ERK through the IL-17 pathway remain unclear. LC3 (microtubule-associated protein 1 light chain 3), an autophagosome marker, is localized in autophagosome membranes after processing [41]. Although the related marker LC3 has been reported in lung cancer [42], gastrointestinal cancers [43], and gliomas [44], the related influence molecules and mechanisms are still unclear. In this study, we confirmed TRAF6 elevated the expression of LC3 and it was essential to accelerating autophagy in gastric cancer cells.

In conclusion, TRAF6 promotes proliferation, migration, and stemness in gastric cancer cells. These results suggested that TRAF6 can be used as a target to provide new strategies for the accurate treatment of gastric cancer.

## Figures and Tables

**Table 1:** siRNA oligonucleotides.

	Forward sequences	Reverse sequences
NC	5′-TTCTCCGAACGTGTCACGT-3′	5′-ACGUGACACGUUCGGAGAATT-3′
siRNA-1	5′-GCAAAUGUCAUCUGUGAAUTT-3′	5′-AUUCACAGAUGACAUUUGCTT-3′
siRNA-2	5′-GCAGUGCAAUGGAAUUUAUTT-3′	5′-AUAAAUUCCAUUGCACUGCTT-3′

**Table 2:** Sequences of primers used in RT-PCR.

	Forward sequences	Reverse sequences
TRAF6	5′-CGCGCACTAGAACGAGCAAG-3′	5′-CAGAACCTATGGCCGCATGG-3′
CK18	5′-CAGGACCTCGCCAAGATCAT-3′	5′-GTTCTCCAAGCTGGCCTTCA-3′
Lgr5	5′-ATGTTCACTGCTGCGATGAC-3′	5′-AGGCTCAAGATGAACGTGAC-3′

## Data Availability

The various raw data and methods used to support the findings of this study are available from the corresponding author upon request.
